# Stress Granules Involved in Formation, Progression and Metastasis of Cancer: A Scoping Review

**DOI:** 10.3389/fcell.2021.745394

**Published:** 2021-09-17

**Authors:** Mohammad Reza Asadi, Dara Rahmanpour, Marziyeh Sadat Moslehian, Hani Sabaie, Mehdi Hassani, Soudeh Ghafouri-Fard, Mohammad Taheri, Maryam Rezazadeh

**Affiliations:** ^1^Molecular Medicine Research Center, Tabriz University of Medical Sciences, Tabriz, Iran; ^2^Student Research Committee, Tabriz University of Medical Sciences, Tabriz, Iran; ^3^Department of Medical Genetics, Faculty of Medicine, Tabriz University of Medical Sciences, Tabriz, Iran; ^4^Student Research Committee, University of Social Welfare and Rehabilitation Sciences, Tehran, Iran; ^5^Department of Medical Genetics, School of Medicine, Shahid Beheshti University of Medical Sciences, Tehran, Iran; ^6^Skull Base Research Center, Loghman Hakim Hospital, Shahid Beheshti University of Medical Sciences, Tehran, Iran

**Keywords:** stress granules, cancer, progression, metastasis, G3BP1, TIA1, TIAR, YB1

## Abstract

The assembly of stress granules (SGs) is a well-known cellular strategy for reducing stress-related damage and promoting cell survival. SGs have become important players in human health, in addition to their fundamental role in the stress response. The critical role of SGs in cancer cells in formation, progression, and metastasis makes sense. Recent researchers have found that several SG components play a role in tumorigenesis and cancer metastasis via tumor-associated signaling pathways and other mechanisms. Gene-ontology analysis revealed the role of these protein components in the structure of SGs. Involvement in the translation process, regulation of mRNA stability, and action in both the cytoplasm and nucleus are among the main features of SG proteins. The present scoping review aimed to consider all studies on the effect of SGs on cancer formation, proliferation, and metastasis and performed based on a six-stage methodology structure and the PRISMA guideline. A systematic search of seven databases for qualified articles was conducted before July 2021. Publications were screened, and quantitative and qualitative analysis was performed on the extracted data. Go analysis was performed on seventy-one SGs protein components. Remarkably G3BP1, TIA1, TIAR, and YB1 have the largest share among the proteins considered in the studies. Altogether, this scoping review tries to demonstrate and provide a comprehensive summary of the role of SGs in the formation, progression, and metastasis of cancer by reviewing all studies.

## Introduction

In 1988, dense cytoplasmic bodies formed under stress in chicken embryonic fibroblasts were named stress granules (SGs) (Collier et al., 1988). SGs are dense bodies that, under stress, are composed of RNA and proteins and are located in the cytosol (Gutierrez-Beltran et al., 2015). Ribonucleoproteins appear under different stresses, and with the end of stress, their number decreases and is limited to SGs being disassembled (Kedersha et al., 2013). Within a specific classification of stresses based on a study in 2008, two categories can be presented: Type I stresses preferentially induce SG formation, which includes hypoxia, heat shock, and arsenic, whereas type II stresses especially activate stress-responsive MAPK cascades, which include X-rays and genotoxic drugs like methyl methanesulphonate (MMS), etoposide (Arimoto et al., 2008). In response to this diversity of stress, the cell pursues an evolutionary strategy that leads to the formation of SGs (Jevtov et al., 2015). Stopping the translation process due to stress builds an extensive repository of components of SGs, translation initiation factors, RNA binding proteins, and non-RNA binding proteins constitute the protein components, and mRNA, which is a non-protein part (Cao et al., 2020). With the release of stress and the end of translational inhibition, the SGs disassemble, and the mRNA makes its way to the translating polysomes (Aulas et al., 2017; Khong and Parker, 2018).

Decision points are a term that can be attributed to SGs even though no specific function has been assigned to them so far (Buchan and Parker, 2009). The decision point for the two components of SGs is the mRNA trapped in their structure and the proteins that make them up (Decker and Parker, 2012). mRNA can take three pathways, remain in the structure of SGs and be stored, resume translation from the structure of released SGs, or move toward degradation. Interestingly, factors such as low translatability, increased coding region length, and untranslated region can also positively increase the likelihood of mRNA being present in the SG structure (Aulas et al., 2017). The major protein component of SGs, which is composed of RNA-binding proteins, can also have two specific domains, Prion Like Domains (PLDs) and Intrinsically Disordered Domains (IDDs), which have the potential to form protein aggregates (Gilks et al., 2004). Low complexity sequence is one of the main features of IDDs and PLDs domains identified by single amino acid repeats with polar residues such as tyrosine, serine, asparagine, and glutamine (Malinovska et al., 2013). These domains can cause SGs to assemble during electrostatic interactions by enhancing the liquid-liquid phase separation (Lin et al., 2015). When protein-overloaded RNAs (especially proteins with IDDs and PLDs domains) dispersed in the cytoplasm or nucleoplasm (soluble phase) coalesce into a concentrated state, liquid-liquid phase separation occurs (condensed phase). During this condensed phase, the highly concentrated RNAs and RNA-binding proteins (RBPs) act as a single organelle with liquid-like properties and high interactions to form SGs (Yang et al., 2020).

Stress granules, with their strong presence, have established themselves in a wide range of diseases, and many studies have shown the extent of these diseases, including cancer (Buchan, 2014), neurodegenerative diseases (Asadi et al., 2021), autoimmune diseases (Johnson et al., 2016), and many other diseases. Among these, cancer can be discussed from three different perspectives: the formation of cancer and tumorigenesis, cancer survival and metastasis, invasion, and progression of cancer cells (Hamidi and Ivaska, 2018). Cancer cells respond to mutant oncogenes by over-proliferation and over-cellular potency, so it makes perfect sense to face more stress (Urra et al., 2016; El-Naggar and Sorensen, 2018). On the other hand, adapting to stresses caused by over-proliferation is also a characteristic of cancer cells (Sharma et al., 2016), which generally in cells with normal conditions lead to death, but in cancerous conditions, the cell quickly adapts survives (Senft and Ronai, 2016). Remarkably, cancer uses the ownership and usability of SGs against stress from the cell to benefit from better tumorigenesis and progression (Grabocka and Bar-Sagi, 2016; Protter and Parker, 2016). Thus, SGs have been introduced as a cancer cell stress-adaptive strategy for a wide range of tumor-related stresses, including proteotoxic stress, oxidative stress, and osmotic stress for the cell (Somasekharan et al., 2015; Grabocka and Bar-Sagi, 2016). In addition to affecting cell proliferation, pro-tumorigenic hyperactivation signaling pathways increase the formation of SGs, which prolongs the life of cancer cells and leads to tumor cell proliferation. On the other hand, the prominent role of SGs in resistance to anticancer drugs is a powerful lever of cancer (Cruz et al., 2019; Herman et al., 2019). So far, many studies have been done on the structure, components, and derivatives of SGs in cancer. As a systematic scoping review, the present study revised all studies on SGs to summarize all aspects of their effects on cancer, from its formation to its progression and metastasis. It also provided a table of eligible studies that included major findings, major methods, and, most importantly, the SGs protein components examined further by gene ontology analysis.

## Materials and Methods

### The Review’s Overall Framework

The method proposed by Arksey and O’Malley (2005) served as the basis for this article’s strategy. Levac et al. (2010) and Colquhoun et al. (2014) later improved on this strategy. The 5-step framework is followed in this review, which includes the following steps, respectively: Classifying the research question, Search plan, Study selection, Data collection, Data summary, and synthesis of results. The sixth and final step is consultation, which is not covered in this article. The Preferred Reporting Items for Systematic Reviews and Meta-Analysis Extension for Scoping Reviews (PRISMA-ScR) Checklist is used to consider and observe two crucial aspects of clarity and transparency while writing the article (Tricco et al., 2018).

### Classifying the Research Question

The overall main research question developed is defined as:

‘What studies have been done on SGs in formation, progression and metastasis in cancer?’
‘What are the results and findings of these studies?’

It should be noted that general and comprehensive questions are considered to include significant studies.

### Search Plan

Pubmed, Embase, Scopus, Cochrane, Google Scholar, Web of Science, and ProQuest were searched to access the publications. A date, language, subject, or publication type restriction was not applied to the search. Review publications were also revised to eliminate the possibility of related articles being ignored. We almost used the following search query for our searches in cancer: “cancer^∗^” OR “neoplasm^∗^” OR “cyst^∗^” OR “carcinoma^∗^” OR “adenocarcinoma^∗^” OR “neurofibroma^∗^” OR “tumor^∗^” OR “tumor^∗^” OR “malign^∗^.” The search keywords and search results in each database are listed in medical subject heading (MeSH) for the PubMed database, and emtree for the Embase database are also used correctly in the search. The last search was led on July 15, 2021. The references were managed using EndNote X8.1.

### Study Selection

Cancer studies involving SGs in humans, cell lines, and animal model studies were screened from the publications found during the search. All types of publications, including journal articles, conference presentations, Erratum, conference abstracts, and reports, were screened. Two reviewers (MA and DR) independently completed the screening in two stages (first only title and abstracts, second full-text). The titles and abstracts of the articles were reviewed at this stage using the Inclusion and Exclusion criteria listed below. The full text of the articles was reviewed, and irrelevant articles were removed, ensuring that the articles were entirely consistent with the research questions. Any discrepancies in agreement with the third person’s opinion were resolved.

#### Inclusion Criteria

I. studies include: SGs in the formation, progression, and metastasis of cancers (any cancer) (all human studies/animal studies/cell culture studies).
II. Articles in English only.
III. Original studies.

#### Exclusion Criteria

I. Studies of SGs in diseases other than cancer.
II. Studies on the effect of SGs on cancer treatment (anticancer medications, chemotherapy, and radiotherapy).
III. Languages other than English.
IV. Studies that are not original.
V. Studies have used bioinformatics and impractical methods to study stress granules.

### Charting the Data

Following the completion of the final articles that answer the research questions, the data-charting was created to organize the study variables using the following headings: author’s name, year of publication, country, type of study, human samples, animal models, cell lines, SGs protein components, methods, major findings, and references. Two reviewers (MA and DR) extracted data from articles using charts separately.

### Data Summary and Synthesis of Results

The findings from the publications presented in tables and charts were subjected to quantitative and qualitative analysis. A descriptive numerical summary of the study’s scope, nature, and distribution was reviewed in the quantitative analysis section. The presented data was affirmed on the broader context suggested by Levac et al. (2010), in a narrative review, in the qualitative analysis section.

## Results

**FIGURE 1:**
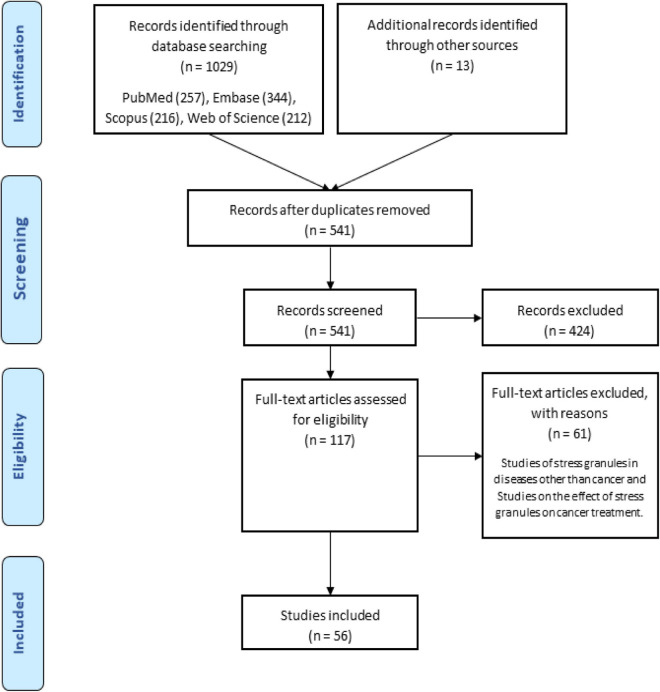
Flow chart of search strategy based on PRISMA flow diagram.

**FIGURE 2:**
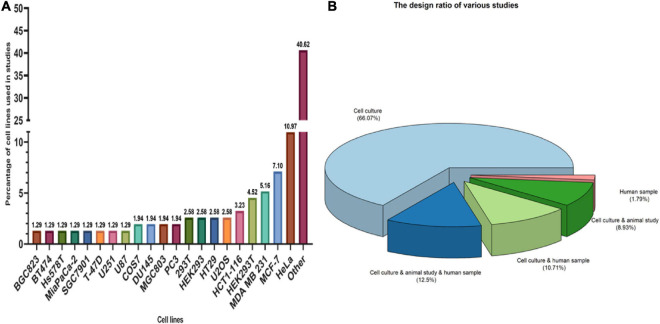
Type of studies and participation of cell lines in studies. **(A)** The longer the bar, the greater the use of cell lines in studies. The figure shows that Hela cells, MCF-7 and MDA MB 231, respectively, had the highest participation among cell lines. OTHER in this section includes cell lines that have been used only once in studies that include: A341, A498, A549, ACHN, 32D mouse progenitor cells, ALL/MIK, AsPC1, Ba-F3/CL1, BCaP, BC-M1, Bladder carcinoma cell line, BxPc3, CAPAN-1, Capan2, Cos-1, CT2A, DG75, DLD1, F470, HCT-8, Hec50, Hepatocellular carcinoma cells (HCC), HFF-1, HGC-27, HPDE, Hs700T, HT-1080, human B lymphoma cells, human PDAC cell line, Jurkat, K562, KrasG12D HPDE cells, Ku812, LAPC4 cell, LC-M1, LNCaP, MCa-PSTC, MKN45, MNNG, MY, Mycoplasma, N2a, NCI-H508, NCI-H747, NCM460, Panc-1, pancreatic cancer cells, PC-E1, RH-30 cells, S2-013, SNB19, SNUC-1, SW480, T98, TOM-1, U118, U343, uroepithelial cell, VCaP, VMRC-LCD, WEHI-3, yeast, and YMB-1. **(B)** Among the types of studies, cell culture studies were more frequent, followed by cell culture, animal study, and human samples studies with the highest number with 12.5% in study design.

**FIGURE 3:**
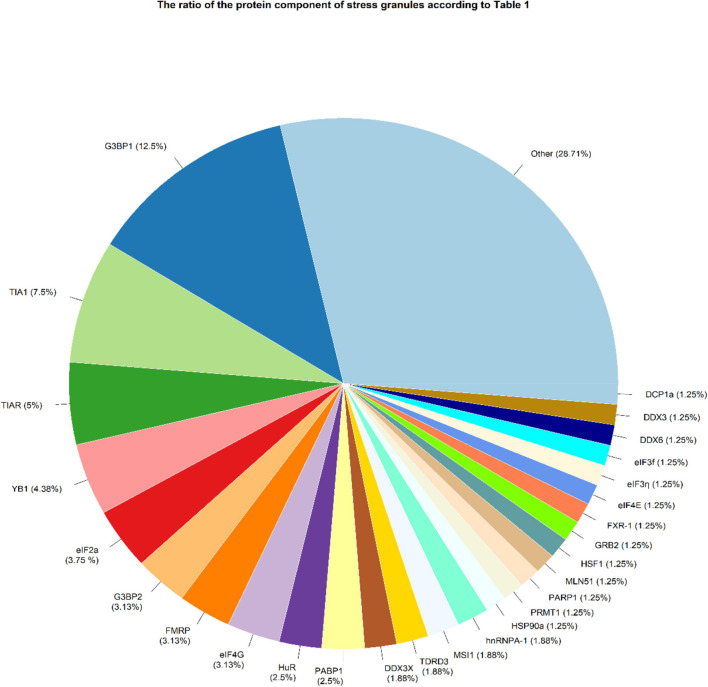
SGs protein components were examined in studies. OTHER refers to proteins that have been considered only once in all studies, including ANG, ASF/SF2, astrin, ATF-4, ATXN2L, CAPRIN1, CIRBP, CRNDEP (peptide), eIF3A, eIF4A1, eIF4D, EWS, FUS, Gal-3, GRP78, hnRNPA/B, hnRNPk, hnRNP-L, HSP70, IGF2BP3, KHSRP, LC3, mTOR, p62, PABP, PABPC1, PRMT7, RACK1, RAPTOR, Rbfox2, RNH1, RPS6, RSK2, S6K1, S6K2, Sam68, SND1, SRSF3, Syk, SYNCRIP, TAF15, USP10, USP9X, and YWHAZ.

There were 1029 results from a keyword search across seven databases. Meanwhile, thirteen additional records were discovered through other sources and added to the total number of articles. Endnote software found and deleted 501 duplicate records, bringing the total number of articles to 541. Following a review of the article titles and abstracts, 117 articles that addressed the research question were chosen. At this point, 56 articles were included in Supplementary Table 1 for the charting data stage after reviewing the full text of 117 articles. Figure 1 shows the step-by-step procedure for selecting eligible articles and studies. Eligible studies were published between 2008 and 2021. Supplementary Table 1 was created to rank studies from top to bottom in order to provide faster access to article division based on study frequency. The percentage of various studies is shown in Figure 2. Meanwhile, the majority of studies, about 66% of studies, are dedicated to cell culture studies only (Baguet et al., 2007; Miyoshi et al., 2007; Arimoto et al., 2008; Eisinger-Mathason et al., 2008; Goulet et al., 2008; Busà et al., 2010; Gottschald et al., 2010; Guo et al., 2010; Kalra et al., 2010; Nikpour et al., 2011; Taniuchi et al., 2011a, b, 2014; Park et al., 2012; Fournier et al., 2013; Pizzo et al., 2013; Thedieck et al., 2013; Chang et al., 2014; Kano et al., 2014; Podszywalow-Bartnicka et al., 2014; Yuan et al., 2014; Krisenko et al., 2015; Liu et al., 2015; Somasekharan et al., 2015; Szafron et al., 2015; Valentin-Vega et al., 2016; Weeks et al., 2016; Narayanan et al., 2017; Wall and Lewis, 2017; Takayama et al., 2018; Haghandish et al., 2019; Heberle et al., 2019; Kashiwagi et al., 2019; Mazloomian et al., 2019; Cui et al., 2020; Do et al., 2020; Brown et al., 2021). After that, cell culture, animal and human sample studies with 12.5% of studies (Grabocka and Bar-Sagi, 2016; Coppin et al., 2017; Wang et al., 2018; Choi et al., 2019; Vellky et al., 2020; Zhang et al., 2021; Zhao et al., 2021), respectively, cell culture and human sample studies with 10% of the total studies (Andersson et al., 2008; Wen et al., 2012; Cougot et al., 2014; Bartkowiak et al., 2015; Chiou et al., 2017; Lin et al., 2019), cell culture and animal study in 9% of the total studies (Meng et al., 2012; Gupta et al., 2017; Morettin et al., 2017; Chen H.-Y. et al., 2018; Wang et al., 2021), had a share of this study. Among these, only one study used just human samples in the study’s design (Zheng et al., 2019). Human cancer samples used in the studies, respectively, include pancreatic cancer sample (Wen et al., 2012; Grabocka and Bar-Sagi, 2016; Coppin et al., 2017), gastric cancer sample (Lin et al., 2019; Zhao et al., 2021), breast cancer sample (Her2 positive or negative) (Cougot et al., 2014; Zhang et al., 2021), prostate cancer sample (Vellky et al., 2020), Renal Cell Carcinoma samples (Wang et al., 2018), Bone marrow aspiration and blood samples (Bartkowiak et al., 2015), and non-small-cell lung carcinoma samples (Zheng et al., 2019). The contribution of cell lines used in the studies is summarized in Figure 2. There are 83, which is given as a bar-plot diagram with the corresponding percentage in Figure 2A; the other in this figure represents cell lines that have only been used once in studies. Figure 3 illustrates the amount of each SGs protein component examined in all studies. The highest rates are related to G3BP1 with 12.5%, TIA1 with 7.5%, TIAR with 5%, and YB1 with 4.5%. Only the most important methods and tests are mentioned due to the large number of methods and tests used in these studies. The distribution of studies is limited to twelve countries, with the United States accounting for the largest share with 13 studies, followed by China and Canada with ten each, Japan with eight, France with three, Netherlands, South Korea, Poland, Germany, and Italy with two each, and Iran and Sweden with one each.

## Discussion

### Cancer, Formation, Progression, and Metastasis

Oncogene activation and tumor suppressor gene (TSG) inactivation can result in uncontrolled cell proliferation, known as cancer (Weiss, 2020). The tumor structure consists of cells that carry changes in the genes that regulate growth and differentiation (Croce, 2008). Oncogenes are involved in the induction of cell proliferation. Changes in these genes can range from developing new oncogenes to the overexpression of common oncogenes that were previously proto-oncogenes. On the other hand, TSGs inhibit cell proliferation by acting in the reverse pathway (Zhu et al., 2015). The features that help cancer cells to progress can be both distinct and complementary and be necessary to the proliferation, survival, and spread of tumor cells which include replication in proliferative signaling pathways, Evasion of growth inhibitors, resistance to programmed cell death, induction of angiogenesis, reprogramming of metabolic mechanisms for anaerobic glycolysis, support for cell proliferation in hypoxia and immune system evasion with The goal to remove these cells in the early stages of progression (Hanahan and Weinberg Robert, 2011).

**FIGURE 4:**
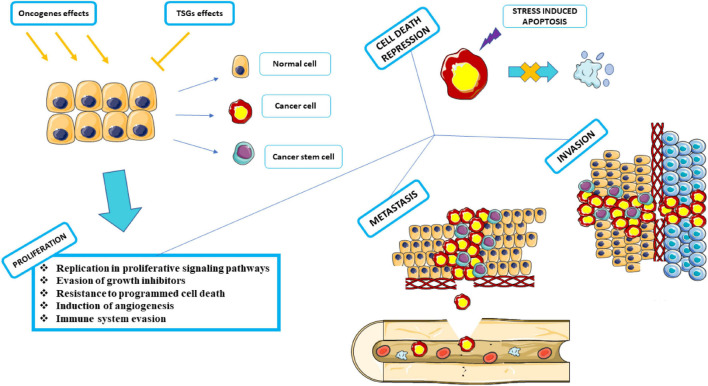
Proliferation, Cell death repression, Metastasis, and Invasion. Under the influence of activated or proliferated oncogenes, cells progress to cancer by inhibiting the activity of tumor suppressor genes. Various factors can act as carcinogens, including chemical carcinogens, physical carcinogens, and oncogenic viruses. Cancer cells must go through several stages in proliferation to arrive at the subsequent phases. Neutralizes apoptosis by inhibiting programmed cell death. It attacks adjacent tissues and expands its dominance by invasion. It metastasizes through the circulatory system and moves from the blood vessels or lymphatic system to various parts of the body, away from the source of its formation, and spreads throughout the organs. This graphical figure was created using the vector image bank of Servier Medical Art (http://smart.servier.com).

Different types of cancers based on cellular origin in a common classification can be divided into four main categories: carcinoma, sarcoma, melanoma, lymphoma, and leukemia (Carbone, 2020). Tumors caused by cancer can be divided into two categories based on their characteristics: malignant or benign (Kalkat et al., 2017). One of the main characteristics of malignant tumors is the ability to metastasize (Tarin, 2011). Metastasis is the ability to enable cancer cells to spread to other parts of the body. Almost all tumors have the potency to metastasize (Brown et al., 2017). The blood and lymphatic system are the two main bases for metastasis, with either required for metastasis (Alitalo and Detmar, 2012). The stages of metastasis can be summarized in the steps of local invasion, intravasation by blood/lymphatic circulation, and extravasation in new tissue and proliferation and angiogenesis, respectively (Figure 4; Shelton et al., 2010). Meanwhile, the stresses that enter the cancer cells push the situation in a direction to dysregulate the equilibrium of SGs and use SGs as an advantage in cancer conditions to benefit cancer cells (Anderson et al., 2015; Do et al., 2020).

### A Precise Glance at Stress Granules: Canonical or Non-canonical Stress Granules

There are two ways to form SGs. The path, which the SGs formation is eIF2α-dependent, and eIF2α is involved in forming SGs leads to the formation of canonical SGs (Bhardwaj et al., 2019). Stress affects eIF2α, mediates serine 51 phosphorylation, and initiates the production of canonical SGs by stopping the development of the translation initiation complex due to lack of GDP / GTP exchange for eIF2α-GTP tRNA-met (Dang et al., 2006). Four stress-related kinases have the ability to phosphorylate the alpha subunit, including PKR under viral infections, PERK due to ER stress, HRI kinase (heme-regulated inhibitor) under osmotic stress and oxidative stress, and GCN2 kinase activated under amino acid starvation (Aulas et al., 2017; Wolozin and Ivanov, 2019). Inhibition of proteasomes that can target these kinases by MG132 and lactacystin can lead to the continuation of these kinases’ effect and the production of canonical SGs (Mazroui et al., 2007).

**FIGURE 5:**
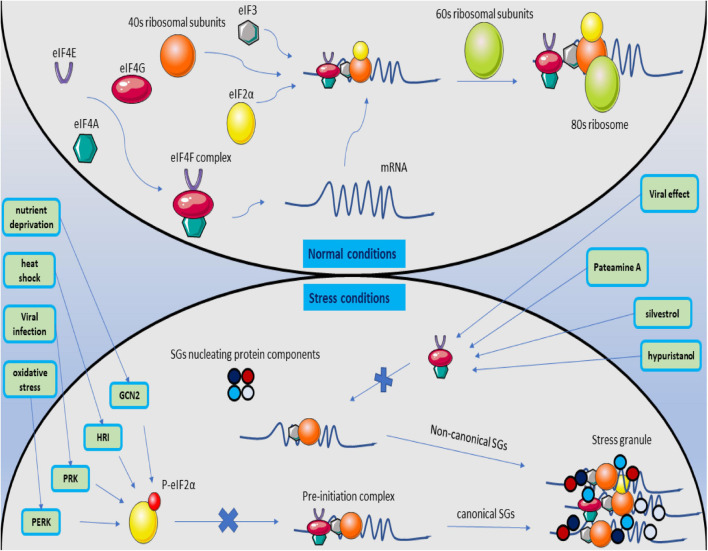
Magnification of canonical and non-canonical stress granules. Under normal conditions, the translation process begins with forming the eIF4F structure and identifying the 5′ cap structure on the mRNA. The pre-initiation complex is formed by joining eIF3, the ribosomal 40S subunit, and non-phosphorylated eIF2a with the initial tRNA. Then, with the 60S ribosome subunit, the ribosome structure is completed, and the translation process is followed. When the cell is stressed, it activates PERK, PRK, HRI, and GCN2 kinases, causes phosphorylation of eIF2a, and prevents the binding of P-eIF2a to the PIC structure. The exact process creates the structure of canonical SGs. If the stress on the cell proceeds by inhibiting the formation of the eIF4F structure by affecting its subunits, the formation of non-canonical SGs is followed by merging the SGs nucleating protein components in the eIF2a independent manner.

eIF4A, eIF4E, and eIF4G are also components of the eIF4F complex, which detects the 5′ cap structure on mRNA, which can, if changed or not appropriately functioned, halt translation at the pre-initiation stage and produce SGs called non-canonical which are entirely independent of eIF2α(Mokas et al., 2009). If any of the eIF4F components are inhibited or interfere with their performance, the translation’s beginning is hindered. Pateamine A, silvestrol, and hypuristanol may result in the production of non-canonical SGs by degrading eIF4A activity (Mokas et al., 2009), affecting eIF4E (Fujimura et al., 2012), and the destructive effect of the virus on the eIF4G structure (Yang et al., 2018; Figure 5).

In general, canonical or noncanonical SGs increase the number of SGs when the cell is under stress and agitated the equilibrium between SGs assembly and disassembly (Hofmann et al., 2021). Among these, by relieving stress, the formed SGs move toward disassembly. The most critical process that stops due to stress and causes SGs to develop is the translation process. Therefore, resuming translation by relieving stress causes SGs to disassemble (Baumann, 2021). Disassembling SGs occurs in several stages, beginning with the RNA leaving the SG structure and entering the suspended translation process. This RNA release is accompanied by structural instability of the SG, leading to the decomposition of a complete SG structure into smaller core structures that continue disassembling or clearance by autophagy, the final number of SGs decreases (Wheeler et al., 2016).

### Stress Granules Assembly Through Cancer Signaling Pathways

Stress that affects cancer cells is not the only cause of SGs formation. Dysregulation of specific signaling pathways associated with inhibition of translation or protein-protein interactions can also induce the formation of SGs (Thedieck et al., 2013; Heberle et al., 2019). The mammalian target of rapamycin (mTOR) is one of the most critical pathways with a considerable contribution in inducing the formation of SGs. mTOR forms two separate complexes, both functionally and structurally, which include mTOR1 and mTOR2. mTOR1 is responsible for regulating cell growth and metabolism, while mTOR2 regulates cell proliferation and survival (Wolfson and Sabatini, 2017; Unni and Arteaga, 2019). mTOR plays an essential role in many signaling pathways, including PI3K/AKT, TSC1/TSC2/Rheb, and AMPK (Dowling et al., 2010). In abnormally activated mTOR tumor cells, it sends growth, metastasis, and invasion signals to other tissues (Hsieh et al., 2012). Among these, up-regulation of the PI3K / AKT / mTOR pathway is one of the main pathways in many malignant tumors (Lim et al., 2015). Assembly of SGs can also be involved in mTORC-related pathways. One of the ways SGs are formed is through the mTORC pathway. On the other hand, inhibition of mTORC1 by Torkinib can lead to a destruction in the formation of SGs, or cell depletion of eIF4G1 or eIF4E, which can neutralize the SG-associated antiapoptotic p21 pathway (Fournier et al., 2013). Raptor is part of the structure of mTORC1, which can be associated with SGs (Takahara and Maeda, 2012). Meanwhile, astrin, as a negative regulator of mTORC1, causes raptor localization in the structure of SGs. This localization inhibits mTORC1 over-activation and inhibits apoptosis (Thedieck et al., 2013). mTORC1 activation mediated by PI3K and P38 hierarchically leads to an increase in the SGs assembling, affecting the raptor (Heberle et al., 2019).

Impressively, it should be noted that SGs and mTORC1 play a role in bilateral regulation. SGs participate in this regulation by incorporating mTORC1 components, including raptor and α, β, and γ subunits (Hofmann et al., 2012; Wippich et al., 2013; Heberle et al., 2019). Conversely, inhibition of mTORC1 is also associated with increased SGs production. mTORC1 inhibits the effect of 4E-BP on eIF4E by phosphorylation and inactivation of eIF4E-BP during the PI3K-mTOR kinase cascade, forming the eIF4F complex, which is responsible for identifying the cap structure at the 5′ mRNA end, thus initiating the translation phase. By inhibiting mTORC1 under stress, eIF4E-BP remains active and inhibits the formation of the eIF4F complex, halting the translation process in the initial stage. This process predisposes the SGs to form by leaving the PIC (pre-initiation complex) on the mRNA and acting as a nest (Frydryskova et al., 2016; Wolozin and Ivanov, 2019). The point to consider is the SGs-mTORC1 interactions, whether the rise or reduction in SG assembly overlaps with the inhibition or activity of mTORC1. Eventually, cancer cells inhibit the conduction of cancer cells to apoptosis by inhibiting hyper-activation of mTORC1 by SGs (Wippich et al., 2013).

RTK-RAS is one of the other essential pathways involved in cancer and is recognized by the cancer genome atlas (TCGA) as the most highly modified oncogenic network in cancer (Sanchez-Vega et al., 2018). twenty to thirty percent of all human cancers have RAS (KRAS-HRAS-NRAS) alteration (Cerami et al., 2012). KRAS is common in pancreatic adenocarcinomas and colorectal cancer, NRAS in melanoma, thyroid cancer, and leukemia (Gao et al., 2013). Cancer cells are under different stresses and must be adapted. However, the mutant RAS protein is the equipment of these cells and equips the cell against tumor-associated stresses to satisfy stress adaptation (Tao et al., 2014; Yang et al., 2018). Remarkably, the presence of SGs was observed in mutant KRAS pancreatic cancer cells as opposed to normal cells. SGs are among the primary responses to stimulation in the survival of mutant KRAS pancreatic cancer cells compared to KRAS-WT cancer cells. KRAS mutants induce the formation of SGs by up-regulating 15-Deoxy-delta (12,14)-prostaglandin J (2) (15d-PGJ2) through downstream effector molecules, RALGDS, and RAF (Grabocka and Bar-Sagi, 2016). 15d-PGJ2 targets cystine 264 in eIF4A, destroying its interaction with eIF4G, the interaction required for the translation process. The effect on this interaction inhibits translation and leads to the formation of SGs (Kim et al., 2007). Instead, mutant KRAS with up-regulation of the nuclear factor erythroid 2-related factor 2 (NRF2) causes rearrangement of glutamine metabolic pathways in tumor cells (Hamada et al., 2021). In addition to its effect on glutamine metabolic pathways, NRF2 is involved in the 15d-PGJ2 effect on the SGs formation (Mukhopadhyay et al., 2020). In the absence of glutamine, an increase in GIRGL LncRNA levels in the cell forms a complex between GLS1 mRNA and CAPRIN1, which induces SGs and inhibits GLS1 mRNA translation by increasing the LLPS process in CAPRIN1, allowing the cancer cell to survive (Wang et al., 2021). Meanwhile, KRAS causes up-regulation of 15d-PGJ2 by increasing the expression of Cyclooxygenase-2 (COX-2). Increasing the levels of 15d-PGJ2 leads to an increase in the assembly of SGs by affecting eIF4A (Qiang et al., 2019). On the other hand, sorafenib, an anticancer medication that increases the production of SGs along the GCN2 / eIF2a pathway, is highly dependent on COX-2 expression. COX-2 is colocalized in the structure of SGs, and inhibition of COX-2 by its inhibitor, celecoxib, results in increased response to sorafenib treatment (Chen W. et al., 2018).

**FIGURE 6:**
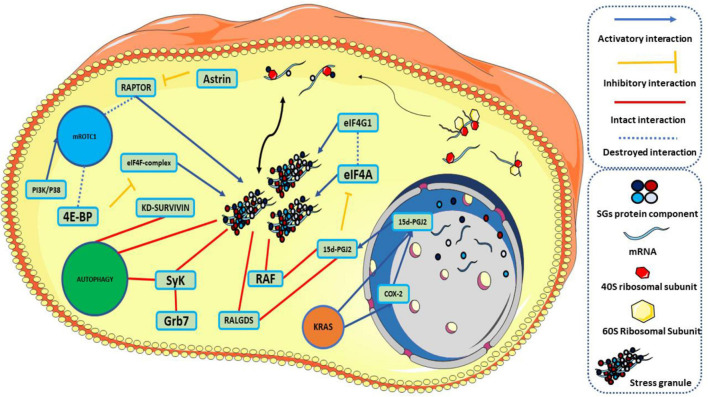
Traces of stress granules in signaling pathways. Under natural conditions, stress granules are assembled and disassembled under an equilibrium. KRAS causes up-regulation of 15d-PGJ2 directly or through COX-2. On one side of the pathway, 15d-PGJ2 interacts with RALGDS and RAF to form SGs. 15d-PGJ2, on the other hand, targets eIF4A, destroys its connection to eIF4G, stops the translation process, and leads to the formation of SGs. Knocking down survivin is associated with an increase in the number of SGs and activation of autophagy. SyK phosphorylation by Grb7 localizes SyK in the structure of SGs and activates autophagy. Localization of RAPTOR in the SGs structure by up-regulation of astrin leads to an increase in the production of SGs. On the other hand, inhibition of mTORC1 inhibits phosphorylation of 4E-BP and the formation of eIF4F complex and causes the formation of SGs. 15d-PGJ2: 15-Deoxy-delta (12,14)-prostaglandin J (2), KD-survivin: knocking Down-Survivin. This graphical figure was created using the vector image bank of Servier Medical Art (http://smart.servier.com).

When the cell experiences different types and many stresses, the involvement of autophagy in stress-responsive mechanisms is inevitable. Autophagy is a metabolic and homeostasis-maintaining intracellular recycling system and cellular self-degradation process that has evolved over time. Autophagy is activated in response to various cellular stresses, such as nutrient deficiency, organelle damage, and abnormal protein accumulation (Mizushima and Levine, 2010). Autophagy inhibits cancer cell survival and induces cell death, suppressing tumorigenesis in cancer cells. Conversely, autophagy can aid tumorigenesis by encouraging cancer cell proliferation and tumor growth (Salminen et al., 2013). survivin is an antiapoptotic protein that inhibits caspase activity (Altieri, 1994). Silencing survivin increases the production of SGs and has the ability to activate the Autophagy signaling pathway as an alternative to survival in hepatocellular carcinoma cells. After the cell is released from stress, autophagy can accompany the cell’s survival (Chang et al., 2014). Syk a cytoplasmic kinase, which depending on the type of cancer cells, can appear on both the anticancer and cancer promoter fronts (Krisenko and Geahlen, 2015). Grb7 phosphorylates syk in the tyrosine residue under stress that induced SGs formation and recruited in the structure of SGs. When the stress is relieved, this recruitment promotes the formation of autophagosomes and the clearance of SGs from the cell, enhancing the cells’ ability to withstand the stress stimulus (Figure 6; Krisenko and Geahlen, 2015).

### Stress Granules Involved in Cancer Characteristics

#### Proliferation

In general, the effect of SGs on proliferation goes through two paths—effect on cell cycle and proliferation regulating factors and effect on transcripts of these factors. SGs play an essential role in keeping cells in the cell cycle progression and preventing cells from entering cell death phases. Specific protein 1 (SP1) is a transcription factor with a significant role in regulating SG-nucleating proteins such as HuR, TIA1 / TIAR, and G3BP1. Interestingly, depleting of the cell of SP1 leads to cell death (Mahboubi and Stochaj, 2017). HuR and CIRP are colocalized in the SGs, and CIRP plays a pivotal role in HUR’s positive regulation. On the other hand, HuR increases the level of cyclin-E1 in breast cancer cells (Guo et al., 2010). When constitutively overexpressed in the mouse mammary gland, Cyclin E1 can act as a true oncogene, driving the formation of tumors, though with low penetrance (10%) and long-latency (Bortner and Rosenberg, 1997). Overexpression of cyclin E1 increases the proportion of cells in the S phase, which leads to increased Rb phosphorylation and cell proliferation in many cancer cell line models (Hwang and Clurman, 2005). Cancer stem cells (CSCs) are a small subset of tumor cells that play a vital role in the proliferation phase due to their reproducibility, differentiation, and tumorigenesis (Samanta and Kar, 2021). Musashi1 (MSI1) is closely related to CSCs with many regulatory interactions as part of the structure of SGs. In colorectal cancer, MSI1 promotes the development of CD44 cancer stem cells (Chiou et al., 2017). MSI1 participates in the PKR/eIF2 cancer stem cell-enhancing machinery and promotes proliferation (Chen H.-Y. et al., 2018). RSK2, one of the protein components of SGs, is released from the structure of SGs under the influence of mitogen and has a direct effect on cyclin D1 and follows by proliferation (Eisinger-Mathason et al., 2008). CRNDEP, a polypeptide produced by the CRNDP gene, is also part of the structure of SGs and is present in highly proliferative tissues with increased expression compared to other tissues (Szafron et al., 2015).

The effect on the transcript of factors and effectors in the proliferation process can be pursued in two ways:

I. The effect of SG components on the transcripts of factors involved in proliferation.
II. Localization of transcripts of factors involved in proliferation as an RNA component in the structure of SGs.

Rbfox2 is a protein responsible for regulating the mRNA stability of many genes and acts as an important member in alternative splicing (Lovci et al., 2013). Rbfox2 in the structure of SGs affecting the mRNA of Rb1, a tumor suppressor, and reducing its stability and expression, increases the proliferation process in cancer cells. Remarkably, by isolating Rbfox2 from the structure of SGs, resveratrol inhibited its effect on Rb1 and effectively reduced the proliferation of cancer cells (Choi et al., 2019). Y-box binding protein-1 (YB-1) is one of the multifunctional proteins that have a role as a regulator in translation and transcription, and by regulating cell cycle progression at G1 / S plays an essential role in the growth and proliferation of tumor cells (Fujiwara-Okada et al., 2013). short RNA antisense to dicer1 (shad1) can be colocalized with YB-1 in the structure of SGs and plays a vital role in regulating the proliferation of cancer cells, including prostate cancer cells, by affecting the expression of YB-1, DLX2, and IGFBP2 (Liu et al., 2015). Impressively, only 15% of all cell mRNAs are colocalized in the SG structure (Khong et al., 2017), which, this mRNA component of SGs through gene enrichment analysis, primarily identified as proto-oncogene transcripts with high enrichment (Namkoong et al., 2018).

MUC4 is an explicit marker of epithelial tumors, and its expression is linked to the degree of differentiation in various cancers. MUC4 has emerged as a specific dysplasia marker expressed in the early dysplastic lesions prior to several malignancies, including incurable pancreatic cancer (Chakraborty et al., 2008). MUC4 mRNA in cancer cells is stabilized by Gal-3, which is found in the structure of hnRNP-L-containing RNA granules. Gal-3 acts as a non-classic RBP in the structure of SGs by interacting with hnRNP-L (Coppin et al., 2017). Under ER stress, BCR-ABL1 mediated TIAR activation. TIAR is a component of cytoplasmic SGs that affects the ARE site in BRCA1 mRNA and can result in its down-regulation in BCR-ABL1 leukemia, which leads to genomic instability. HuR influenced BRCA1 translation and mRNA stability positively (Podszywalow-Bartnicka et al., 2014). It should be noted that the oncogenic tyrosine kinase BCR-ABL is also localized in the structure of SGs, and the formation of granular structure is necessary for the activity of ABL kinase and N-terminal region of BCR (Kashiwagi et al., 2019).

#### Cell Death Repression

Many studies have examined the role of SGs in Cell death repression and inhibition of apoptosis, and the vital role of SGs in these processes has been well established. According to studies in Cell death repression, SGs also use their structural capacity and change the cell’s fate by including essential components in this pathway. Among these, SGs are involved in the sequestration of pro-apoptotic proteins and the inclusion of mRNAs of important apoptotic mediators and their protection and involvement in the regulatory mechanisms of reactive oxygen species (ROS), which are further reviewed in this study. Raptor sequestration, part of the mTORC1 structure in the structure of SGs due to astrin and its role in inhibiting apoptosis, was mentioned (Thedieck et al., 2013). S6K1 and S6K2 are among the influential factors on mTORC1. S6K1 under mild arsenite stress and S6K2 under mild and acute arsenite stress are localized in the structure of SGs. RSKS1, the ortholog of S6K1 and S6K2 in C.eleganse, localized under stress in the structure of SGs and hindered apoptosis through inhibiting mTORC1 hyper-activation (Sfakianos et al., 2018).

Under oxidative stress conditions, RACK1 interaction is inhibited by localization in the structure of SGs with MTK, a MAPK kinase required for apoptosis due to P38 / JNK activation (Arimoto et al., 2008). MSI1 is up-regulated as SGs component in bladder carcinoma cell lines relative to normal uroepithelial cells and inhibits apoptosis by targeting mRNA of essential genes, including P21 ^*CIP*1^(Nikpour et al., 2011). On the other hand, Macrophage-inhibitory cytokine-1 (MIC1), one of the pro-apoptotic proteins associated with the pathogenesis of many cancers under ER stress, activates the ERK1 / 2 signaling pathway, which stabilizes MIC1 mRNA in the structure of SGs (Park et al., 2012). USP9X acts as one of the most critical proteins in inhibiting apoptosis (Kushwaha et al., 2015). Ubiquitin Specific Peptidase 9 X-Linked (USP9X) and Tudor Domain Containing 3 (TDRD3) are colocalized in the structure of SGs. The presence of TDRD3 is essential to protect USP9X against de-ubiquitination. Knockdown TDRD3 inhibits the presence of USP9X in the structure of SGs and increases cellular apoptosis (Narayanan et al., 2017). Meanwhile, Increased expression of USP10 in prostate cancer cells in interaction with G3BP2 inhibits the P53 signaling pathway and causes a specific carcinogenic effect along the USP10 / G3BP2 / P53 pathway (Takayama et al., 2018).

#### Metastasis and Invasion

The distinguishing feature between invasion and metastasis is the ability of the metastasis to use the circulatory system or lymphatic system, which spreads cancer cells to tissues farther from the source. In contrast, invasion is defined as the penetration of cancer cells into neighboring tissues (Krakhmal et al., 2015). Metastasis is also a response to the stress that cancer cells undergo, and SGs are used as cellular equipment in this response. SGs allow healthy cells to stop their translation process under stress and keep important mRNAs in the SG structure intact. The exact process in cancer cells gives survival under challenging conditions and paves the way for the later stages of cancer. Remarkably, cells with a higher potential for metastasis also carry more SGs (Somasekharan et al., 2015).

Targeting the formation of SGs by drug inhibition through the NRF2 transcription factor destroys the invasive and metastatic capacity of pediatric sarcoma to the extent that targeting SGs is suggested as a treatment for pediatric brain tumors (Delaidelli et al., 2018). TDRED3, in addition to its role in inhibiting apoptosis by interacting with USP9X (Narayanan et al., 2017), also plays a vital role in determining the invasive capacity of breast cancer cells. Undergoing chemotherapy, TDRD3 is targeted at the structure of SGs. CELL DEPLETION TDRD3 inhibits the progression and invasion of cancer cells (Morettin et al., 2017). On the other hand, increased invasion of gastric cancer cells can occur in response to oxaliplatin through ATXN2L up-regulation, known as the regulator of SGs, with the effect of EGF along with the PI3 / AKT signaling pathway (Lin et al., 2019). G3BP1, as a nucleator member in the structure of SGs, is responsible for a significant part of the SG-dependent metastasis and invasion process (Taniuchi et al., 2011a, b, 2014; Somasekharan et al., 2015; Wang et al., 2018). RAS-GTPase-activating protein SH3 domain-binding protein 1 is overexpressed in many head, neck, prostate, breast, and colon tumors (Xiong et al., 2019). G3BP1 also causes tumor progression and metastasis in renal cell carcinoma cells by over-expression along the IL6 / G3BP1 / STAT3 pathway (Wang et al., 2018). Remarkably, the reduction of G3BP1 levels through YB-1 globally acetylation by MS-275 treatment reduces the sarcoma metastasis and reduces the premetastatic activity of the G3BP1 factor (El-Naggar and Sorensen, 2018). In addition, non-small cell lung cancer patients with clinical stages II and III had higher G3BP1 and YB1 protein expression than those with stage I. Furthermore, G3BP1 protein expression was positively correlated with YB1 and pAKT (Zheng et al., 2019).

Binder of Arl Two (BART) is also one of the main factors regulating and reducing metastasis and invasion of pancreatic cancer cells. The N-terminal part of G3BP can down-regulate BART post-transcriptionally and increases metastatic activity (Taniuchi et al., 2011b). CD24 regulates G3BP endoribonuclease activity and its effect on BART, so the CD24 / G3BP / BART pathway is essential in metastasis (Taniuchi et al., 2011a).

#### Chemotherapy Resistance

Another aspect that cancer cells use to benefit from SGs is the response to treatment and chemotherapy. The equilibrium between SGs assembling and disassembling versus chemotherapy in cancer cells is entangled, and this disequilibrium tends to increase the number of SGs. The common denominator of most chemotherapeutic agents is summarized in eIF2α phosphorylation (Gao et al., 2019). Four stress-associated kinases are considered to phosphorylate eIF2α (Aulas et al., 2017; Wolozin and Ivanov, 2019). Sorafenib and bortezomib are two FDA-approved drugs that are each used to treat specific cancers. Sorafenib is a Raf1 / Mek / Erk kinase inhibitor used to treat hepatocellular carcinoma (Adjibade et al., 2015), thyroid carcinoma (Lin et al., 2021), and renal carcinoma (Chen W. et al., 2018). Remarkably, treatment with sorafenib induces phosphorylation of the eIF2 alpha subunit by PERK and increases the formation of SGs (Adjibade et al., 2015). On the other hand, bortezomib, which is used for the chemotherapy of multiple myeloma, also induces eIF2α phosphorylation by HRI and follows an increase in the number of SGs (Fournier et al., 2010). Bortezomib-induced SGs move in the opposite direction of the effect of bortezomib as an anticancer drug by increasing the degradation of p21 transcripts that play a role in increasing apoptosis (Gareau et al., 2011).

Among the chemotherapeutic agents, there are many cases in which phosphorylates eIF2α through a specific kinase, including sorafenib (Adjibade et al., 2015), lapatinib (Adjibade et al., 2020), arsenite (Zou et al., 2012), thapsigargin (Doan et al., 2015) via PERK, 5-Fu via PKR (Longley et al., 2003), MG132 via GCN2 (Mazroui et al., 2007), and bortezomib via HRI (Schewe and Aguirre-Ghiso, 2009). Increased production of SGs is accompanied by an increase in mechanisms that can resist chemotherapy, including the regulation of apoptosis and autophagy (Chang et al., 2014), the facilitation of ABC family expression (Unworth et al., 2010), and the regulation of malignant cell stemness (Chiou et al., 2017). Meanwhile, according to studies, targeting SGs as anti-stress granule therapy along with conventional chemotherapy can create a new perspective for cancer treatment and has the potential to be recognized through further studies as a new treatment.

### Gene-Ontology Analysis of Stress Granules Protein Components

**FIGURE 7:**
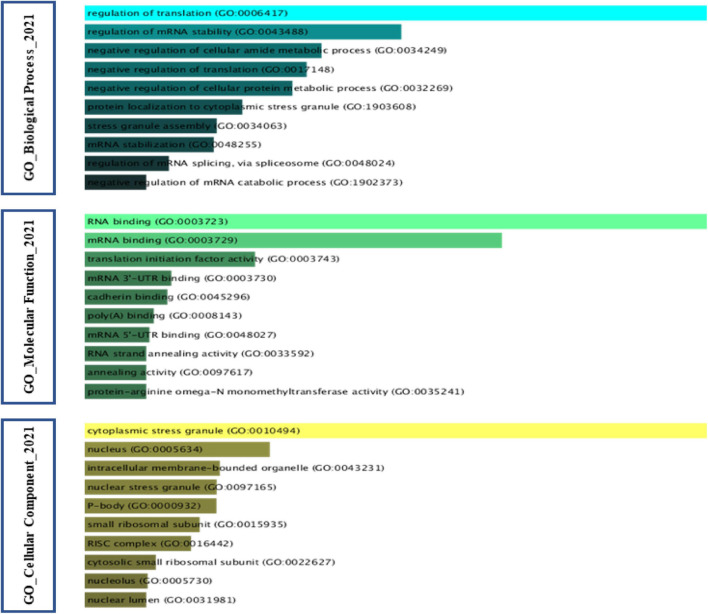
Gene Ontology analysis of Stress Granules protein components in cancer. Gene Ontology analysis was performed on SGs protein components in cancer based on the Table 1. The GO cellular component found that most of the proteins embedded in the structure of SGs exhibit their function in both the cytoplasm and the nucleus. According to GO biological process, the impact on the stability of transcript of factors involved in cancer cell proliferation is one of the networks in which SGs can intervene. GO molecular function analysis further considers a feature such as RNA binding to the protein component of SGs.

A noteworthy point at the end is that gene-ontology analysis of the proteins of SGs that have been extracted based on the studies in Table 1. Regulation of translation has the maximum rate of the physiological function of these proteins concerning other proteins in a biological network. Bring to an end in the translation initiation stage is the central mechanism that underlies the formation of SGs. Likewise, one of the capabilities that SGs provide to cancer cells is the protection or inclusion of transcription factors involved in proliferation as part of their constituent structure, which is confirmed by the GO-biological process. Interestingly, these proteins also play a crucial role in the regulation of mRNA stability by these factors. On the other hand, the function of these proteins must be in the direction of the duty they perform, which has so far been closely related to the RNA molecule. The GO-Molecular function confirmed RNA binding in various forms, including RNA binding, mRNA binding, and mRNA 3′ and 5′-UTR binding. According to the GO cellular component, most of the proteins embedded in the SGs structure have functions in both the cytoplasm and the nucleus (Figure 7).

**TABLE 1:** Stress classifications inducing canonical SGs assembly.

**Stress characteristics**	**SGs inducing target**	**References**
oxidative stress	eIF2α(PERK)	Palangi et al., 2017
ER stress	eIF2α(PERK)	Wang et al., 2019
Viral infection	eIF2α(PKR)	García et al., 2006
nutrient deprivation	eIF2α(GCN2)	Wek et al., 1995
UV irradiation	eIF2α(GCN2)	Deng et al., 2002
heat shock	eIF2α(HRI)	Lu et al., 2001

## Conclusion

Stress granules have become one of the main instruments of cancer cells to deal with stress. Due to their structural capacities, SGs provide cancer cells with the ability to go through the most critical stages in their process. The role of mTOR and RAS pathways in cancer has been proven in many studies. The involvement of SGs and playing a pivotal role in these pathways in different cancers are identified as a common point. On the other hand, the effect of SGs on cell cycle regulating factors and essential factors involved in proliferation in cancer cells is used as a biased mechanism. Utilizing the capacities of SGs in the process of cell death repression and the presence of more SGs in cells prone to metastasis accompanies cancer in the following essential phases. There have been many studies on SGs in cancer formation, progression, and metastasis. In this study, the aim was to provide a comprehensive review to conclude this matter. Overall, this study could pave the way for further studies on SGs in cancers and provide a roadmap to guide these studies.

## Author Contributions

MT, MR, and SG-F wrote the draft and revised it. MA, HS, DR, MM, and MH collected the data and designed the tables and figures. All authors read and approved the submitted version.

## Conflict of Interest

The authors declare that the research was conducted in the absence of any commercial or financial relationships that could be construed as a potential conflict of interest.

## Publisher’s Note

All claims expressed in this article are solely those of the authors and do not necessarily represent those of their affiliated organizations, or those of the publisher, the editors and the reviewers. Any product that may be evaluated in this article, or claim that may be made by its manufacturer, is not guaranteed or endorsed by the publisher.
