# Supplementary material for: Stress Granules Involved in Formation, Progression and Metastasis of Cancer: A Scoping Review
Source: Front Cell Dev Biol. 2021 Sep 17;9:745394. doi: 10.3389/fcell.2021.745394 (PMC8485071; doi:10.3389/fcell.2021.745394)
Supplement: Supplementary file 1 [file Table_1.docx]

| **Author(s) Year of Publication** | **Country** | **Type of study** | **Human sample(s)** | **Animal model(s)** | **Cell line(s)** | **Stress granules protein components** | **Major Method(s)** | **Major findings** |
| --- | --- | --- | --- | --- | --- | --- | --- | --- |
| **Baguet et al. 2007** | **France** | **Cell culture** | **-** | **-** | **HeLa cells** | **TIA1**  **MLN51**  **FMRP**  **PABP1**  **eIF2α** | **Immunofluorescence, FISH, IHC, Western blotting, Fluorescence recovery after photobleaching (FRAP), Cell viability assay** | **MLN51 is localized in the structure of SGs along with FMRP and PABP in human breast carcinoma and tumor structure.** |
| **Miyoshi et al. 2007** | **Japan** | **Cell culture** | **-** | **-** | **HeLa cells**  **Jurkat**  **MCF-7**  **YMB-1**  **HEK-293T** | **HSF1** | **qPCR, Immunoblotting, Immunofluorescence, Ribosome analysis, Pulse-chase analysis of rRNA processing, Effects of RNAi on cell proliferation** | **Bystin increases the proliferation of cancer cells by facilitating the formation of ribosome structure, specifically the 40S subunit.**  **Bystin is placed in the nucleus in a structure other than SGs.** |
| **Andersson et al. 2008** | **Sweden** | **Cell culture**  **Tissue specimen** | **Human normal organ tissue arrays** | **-** | **HT-1080 fibrosarcoma cells**  **F470 primary**  **human fibroblasts** | **FUS**  **EWS**  **TAF15** | **IHC, Differentiation, and proliferation assays, Western blot,** **qPCR** | **FET proteins, including FUS, EWS, and TAF15, are localized in the structure of SGs and have an essential effect on the stress response, regulation of translation, and adhesion, which play an essential role in cancer progression.** |
| **Arimoto et al. 2008** | **Japan** | **Cell culture** | **-** | **-** | **COS-7**  **HeLa cells**  **HEK293**  **DU145** | **RACK1**  **eIF4E**  **eIF4G**  **TIA1** | **Mass-spectrometric analyses,** **Co-immunoprecipitation assay for protein binding,** **Immunoblotting analyses,** **Immunofluorescence staining, and microscopic observation of SGs** | **Type 1 stress includes hypoxia, heat shock, arsenite, and type 2 stress, including x-rays and genotoxic drugs.**  **Type 1 stress causes the formation of SGs. Type 2 stress can induce apoptosis through the P38 and JNK-MAPK pathways.**  **RACK1 is located in the SG structure due to type 1 stress and MTK1-SAPK activation is inhibited, and apoptosis is induced due to type 2 stress.** |
| **Eisinger-Mathason et al. 2008** | **USA** | **Cell Culture** | **-** | **-** | **MCF-7** | **RSK2**  **TIA1** | **Immunodetection,** **qPCR,** **Bromodeoxyuridine Incorporation Assay, IHC, In Vitro Binding Assay,** **Protein Turnover Measurements** | **RSK2 and TIA1 are colocalized in the structure of SGs.**  **Knocking down RSK2 reduces cell survival in response to stress.**  **Mitogen releases RSK2 from the structure of SGs, which enters the cell nucleus and increases cell proliferation through cyclin D1.** |
| **Goulet et al. 2008** | **Canada** | **Cell culture** | **-** | **-** | **HeLa cells** | **TDRD3**  **TIA1**  **FMRP**  **RPS6** | **Immunoblotting, Cell treatments and Immunofluorescence, Protein purification, Peptide binding assay,**  **Pulldowns, and mass spectrometry** | **TDRD3, along with SERPINE1 and DEAD / H box3, are part of the structure of SGs in response to stress. TDRD3 is overexpressed in breast cancer.** |
| **Busa et al. 2010** | **Italy** | **Cell culture** | **-** | **-** | **HeLa cells**  **PC3** | **Sam68**  **TIA1**  **hnRNP A1**  **ASF/SF2** | **Cell proliferation assay, cell viability, and cell cycle**  **Analysis, Stable Knockdown of Sam68 in PC3 cells, Cell transfections and CD44v5-luciferase splicing assay, q-PCR, Western blot, Immunofluorescence, and bromo-uridine incorporation** | **DNA damage causes localization of sam68 in the structure of SGs within the nucleus.**  **sam68 is up-regulated in prostate cancer and promotes resistance to genotoxic stress.**  **Mitoxantrone-induced nuclear stress also follows the localization of sam68 in the structure of SGs and affects its target splicing, CD44.** |
| **Gottschald et al. 2010** | **Germany** | **Cell culture** | **-** | **-** | **A549**  **PC3**  **DU145** | **TIA1**  **TIAR**  **eIF3η** | **Immunocyto- and immunohistofluorescent**  **Analyses, Western blot analysis, q-PCR** | **TIA1 and TIAR reduce HIF1-α expression under conditions of severe hypoxia.** |
| **Guo et al. 2010** | **USA** | **Cell Culture** | **-** | **-** | **MCF-7**  **T47D cells** | **CIRBP**  **HuR** | **Immunoblot Analysis,** **qPCR,** **GST-Pull Down and Immunoprecipitation Assays,** **Gel Shift and UV-Crosslink Analyses,** I**mmunofluorescence Microscopy** | **Cyclin-E1, HuR, and CIRP interact together, and HuR and CIRP are colocalized together in the structure of SGs.**  **CIRP is involved in the positive regulation of HuR.**  **HuR increases levels of cyclin-E1 in breast cancer cells.** |
| **Kalra et al. 2010** | **Canada** | **cell culture** | **-** | **-** | **Mycoplasma** | **YB-1** | **western blot,** **Immunofluorescent imaging,** **Dual-luciferase reporter assay,** **PCR** | **Inhibition of Integrin Linked Kinase (ILK) activity by suppressing TWIST causes down-regulation of YB-1.**  **YB-1 is included in the structure of SGs and regulates Her2 / neu expression.**  **Treatment with ILK inhibitors may be an excellent route to affect Her2 / neu positive tumors.** |
| **Nikpour et al. 2010** | **Iran** | **cell culture** | **-** | **-** | **Bladder carcinoma cell line**  **uroepithelial cell** | **MSI1** | **Methylation analysis, Caspase assays,** **q-PCR,** **Microarray analysis and evaluation,** **Western blotting,** **Immunofluorescence detection of stress granules** | **Musashi1 (MSI1) plays a vital role in apoptosis and cell death by affecting many signaling pathways, specifically p21 in apoptosis, p27 in the cell cycle, and jagged1 in the notch-delta pathway.**  **In general, as a critical element of SGs, it also plays a role in tumorigenesis.** |
| **Taniuchi et al. 2011** | **Japan** | **cell culture** | **-** | **-** | **COS7** | **G3BP1** | **Identification of the BART-binding region in G3BP, q-PCR,** **Matrigel invasion assay** | **The C-terminal portion of G3BP is involved in the phosphorylation of eIF2α and can trigger the formation of SGs.**  **The N-terminal portion of G3BP plays a vital role in the motility and invasion of pancreatic cancer cells by regulating the expression of many genes, including Down-regulation of BART through post-transcriptional regulation and transcription factors and matrix metalloproteinases.**  **BART is a significant factor in inhibiting the retroperitoneal invasion and hepatic metastasis of pancreatic cancer cells.** |
| **Taniuchi et al. 2011** | **Japan** | **cell culture** | **-** | **-** | **S2-013** | **TIA1**  **G3BP1** | **Confocal immunofluorescence microscopy, In vivo binding of CD24 with G3BP, Identification of G3BP RNA ligands, Affinity precipitation of G3BP using the biotinylated**  **BART transcript, In vitro mRNA decay assay/** | **CD24 inhibits the invasion and metastasis of cancer cells by post-transcriptional modification of BART through inhibiting G3BP endoribonuclease activity.** |
| **Meng et al. 2012** | **USA** | **Cell culture**  **Animal study** | **-** | **athymic NCr-NU/NU female mice** | **Hec50 cells** | **G3BP1**  **SND1** | **Immunostaining,**  **MTDH Truncations,**  **Cell Viability,** **MTDH protein coimmunoprecipitation assays,**  **Pulldown of MTDH-associated RNAs,**  **In Vivo Tumor Formation** | **MTDH (cytoplasmic metadherin) is elevated in many solid tumors and is involved in metastasis and chemoresistance.**  **Cytoplasmic depletion of MTDH increases the production of SGs, increasing sensitivity and decreasing cell survival against chemotherapy.** |
| **Park et al. 2012** | **South Korea** | **Cell culture** | **-** | **-** | **HCT-116**  **HT-29**  **HCT-8** | **HuR**  **eIF2α** | **Assessment of Apoptosis by Fluorescence-activated Cell Sorting,** **Western blot,** **q-PCR,** **Luciferase Assay,** **Confocal Microscopy,** **RNA-FISH, Cell Survival Analysis,** **RNA-Chromatin Immunoprecipitation** | **MIC1, a pro-apoptotic protein that is associated with the pathogenesis of many cancers. One of the mechanisms of its mRNA stabilization occurs under ER stress, which activates ERK1 / 2, and this activation prolongs the retention of MIC-1 mRNA by the SG structure.** |
| **Wen et al. 2012** | **USA** | **Cell culture**  **tissue specimens** | **pancreatic cancer tissue** | **-** | **BxPc3**  **MiaPaCa-2**  **HPDE cells**  **KrasG12D HPDE cells**  **HFF-1 cells** | **eIF3f**  **hnRNP k**  **eIF4G** | **Bicistronic luciferase reporter assay,** **q-PCR, Colony assay and soft agar assay, Cell survival, apoptosis, and cell cycle assay, 3D culture of HPDE cells, Knockdown of endogenous eIF3f and hnRNP K,** **Immunoprecipitation and Western blotting,** **IHC** | **rRNA levels Increased in cancer cells and can promote cancer.**  **eIF3f reduces** **rRNA protection by inhibiting the binding of hnRNP k to**  **rRNA and decrease its levels in cells in non-SG foci.** |
| **Pizzo et al. 2013** | **Italy** | **Cell culture** | **-** | **-** | **HeLa cell**  **LNCaP cell** | **ANG**  **RNH1**  **PABP1**  **TIA1** | **Gel filtration chromatography, Sucrose gradient ultracentrifugation, Immunofluorescence and confocal microscopy, FRET, RNH1 knockdown, EB and AO staining of apoptotic cells** | **Under stress, ANG is embedded in the structure of SGs and mediates the production of tRNA-derived stress-induced small RNA (ti-RNA), which alters the cell translation process for survival.**  **The structure of SGs prevents the inhibitory activity of RNH1 on ANG under stress.** |
| **Fournier et al. 2013** | **Canada** | **Cell culture** | **-** | **-** | **HeLa cells**  **MCF-7**  **Hs578T**  **N2a** | **eIF2α**  **mTOR**  **RAPTOR**  **FMRP**  **FXR-1**  **G3BP1** | **q-PCR, Cap-binding assays, Annexin V-FITC, PI assay and FACS analysis, CAM tumor assay** | **In cancer cells, pp242 can inhibit mTOR or deplete the cell of eIF4E or eIF4G1 and inhibit the formation of SGs.**  **As a result, the p21 antiapoptotic pathway is neutralized, and cancer cells become sensitive to chemotherapy and eventually death.** |
| **Thedieck et al. 2013** | **Netherlands** | **Cell culture** | **-** | **-** | **HeLa cells**  **BT474**  **MDA-MB-231**  **MDA-MB-453**  **T-47D**  **MCF-7** | **DDX6**  **PARP1**  **eIF3A**  **ATF-4**  **hnRNP-A1**  **YB-1**  **HSP90**  **HSP70**  **HSF1**  **G3BP1**  **astrin** | **Mass Spectrometry, IP, Immunoblotting, IF, Fluorescence Microscopy, Confocal Microscopy** | **In tumors with up-regulated astrin, inhibition of mTORC1 occurs and causes it to localize in the SG structure(raptor). Increasing the number of SGs and inhibiting mTORC1 inhibits apoptosis in cancer cells.** |
| **Kano et al. 2013** | **Japan** | **Cell culture** | **-** | **-** | **HCT116** | **TIAR**  **SRSF3** | **q-PCR,** **Inhibition of NMD activity and chemiluminescence-based NMD**  **Assay, Western blotting, IHC,** **Promoter activity assay,** **Electrophoretic mobility shift assay,** **Chromatin immunoprecipitation assay** | **Treatment Arsenite, as a hypoxic stress simulator by inhibiting NMD activity, increases the Premature Termination Codon mRNA of SRSF3 protein and converts it to SRSF3-TR upon truncation.**  **SRSF3-TR accumulates in the structure of SGs in the cytoplasm, which triggers an oxidative stress-initiated inflammatory response by regulating IL8 in colon cancer cells.** |
| **Chang et al. 2014** | **China** | **Cell culture** | **-** | **-** | **Hepatocellular carcinoma cells (HCC)** | **LC3**  **p62** | **q-PCR, Western blot, Knockdown of survivin in HCC cells, Cell viability assay, Acridine orange staining for autophagy detection, Electron microscopy, Flow cytometry-based cell-cycle analysis** | **Knocking down of survivin increases the number of SGs, activates one of the autophagy signaling pathways that act as an alternative survival pathway in HCC cells.** |
| **Cougot et al. 2014** | **France** | **Cell culture**  **Human cell sample** | **two non-malignant breast cells**  **three HER2-negative and five HER2-positive breast cancer cells** | **-** | **HeLa cells** | **MLN51**  **Dcp1a** | **FISH, Immunofluorescence on HER2+ and breast cells, IHC,** **Live cell imaging,** **High pressure freezing, freeze substitution, Immunoelectron microscopy** | **MLN51 is located in the structure of SGs and p-bodies in the cytoplasm. Its over-expression in breast cancer cells showed a new localization in the cytoplasm, associated with a decrease in the number of p-bodies.** |
| **Podszywalow-Bartnicka et al. 2014** | **Poland** | **Cell culture** | **-** | **-** | **32D mouse progenitor cells** | **TIAR**  **HuR** | **Western Blotting, Immunoprecipitation,** **RNA isolation and real-time PCR,** **Luciferase activity assay,** **Site-directed mutagenesis,** **Immunostaining,** **Determination of RNA synthesis rate by flow cytometry** | **BCR-ABL1 mediated activating TIAR under ER stress.**  **TIAR is a part of cytoplasmic SGs, affects the ARE site in BRCA1 mRNA, and can cause down-regulation in BCR-ABL1 leukemia, leading to genomic instability.**  **HuR positively regulated BRCA1 translation and its mRNA stability.** |
| **Taniuchi et al. 2014** | **Japan** | **Cell culture** | **-** | **-** | **human PDAC cell line** | **IGF2BP3**  **G3BP1** | **Confocal Immunofluorescence Microscopy/** **Transwell Motility Assay/** **Matrigel Invasion Assay/** **Immunoprecipitation/** **Microtubule Precipitation Assay/** **Immunofluorescence with RNA Fluorescence**  **In Situ Hybridization/** | **KIF20A increased invasion of pancreatic cancer cells by transmitting IGF2BP3 transcripts through microtubules.**  **The IGF2BP3 protein accumulates in the structure of cytoplasmic SGs.** |
| **Yuan et al. 2014** | **China** | **Cell culture** | **-** | **-** | **MDA-MB-231**  **SW480 cells** | **PABPC1**  **KHSRP**  **YBX1**  **SYNCRIP**  **EIF4A1**  **DDX3**  **ELAVL1**  **EIF3F** | **Immunoprecipitation of the MAEL protein complex, Anti-tag coimmunoprecipitation, Immunofluorescence staining of SGs** | **MAEL is aberrantly expressed in many cancers in the structure of SGs and Nuage and may be involved in silencing miRNA in cancer cells.** |
| **Bartkowiak et al. 2015** | **Germany** | **Human sample**  **Cell culture** | **Bone marrow aspiration**  **Blood samples** | **-** | **BC-M1**  **LC-M1**  **PC-E1** | **GRP78** | **Western blot and densitometric analysis, immunohistochemical staining of organ metastases, Grp78 tissue microarrays** | **GRP78 has been observed as a representative of Unfolded Protein Responses (UPRs) in the structure of SGs in Disseminated tumor cells (DTCs).**  **UPRs are activated in DTCs, and their association in the SG structure can be a predictive marker.** |
| **Krisenko et al. 2015** | **USA** | **Cell culture** | **-** | **-** | **HEK293T**  **MCF-7**  **DG75**  **human B lymphoma cells** | **Syk**  **TIAR**  **G3BP1** | **Co-immunoprecipitation assays, Stress granule formation and clearance, Protein interaction screens** | **Syk interacts with the protein component of SGs and is incorporated into the SGs structure via Grb7 and is involved in clearing cells of SGs during autophagy.** |
| **Liu et al. 2015** | **USA** | **Cell culture** | **-** | **-** | **PC-3**  **HeLa**  **HEK293**  **MCF-7**  **COS7** | **YB-1**  **hnRNP A/B** | **RNA immunoprecipitation, RNA sequencing, Genomic analysis, RNA electromobility shift assay, Northern blots and qRT-PCR, Dual fluorescent immunocytochemistry and in situ**  **Hybridization** | **short RNA antisense to dicer1 (shad1) and YB1 colocalize in the structure of p-bodies and SGs.**  **shad1 can play an essential role in prostate cell proliferation by affecting the expression of YB1, DLX2, and IGFBP2.** |
| **Somasekharan et al. 2015** | **Canada** | **Cell culture** | **-** | **-** | **U2OS cells**  **DU-145**  **MNNG cells**  **RH-30 cells** | **YB-1**  **G3BP1**  **GRB2**  **TIA1**  **TIAR**  **FMRP**  **FXR1**  **eIF3η**  **DDX6** | **apoptosis assays, Quantification of SGs, lung metastases, necrosis, and Ki67 staining,** **Immunoblot analysis, riboimmunoprecipitation (RIP), and RT-PCR,**  **Luciferase reporter assays,**  **EMSA,** **In vivo murine renal subcapsular implantation model, Protein carbonyl content measurement, RNA affinity chromatography, Survival analyses** | **YB-1 activates the expression of G3BP1 by acting on its mRNA.**  **The expression of YB-1 and G3BP1 in sarcoma are highly correlated, and elevated expression of G3BP1 as a nucleator in SGs is associated with low survival.** |
| **Szafron et al. 2015** | **Poland** | **Cell culture** | **-** | **-** | **HeLa cells** | **TIA1**  **CRNDEP(peptide)** | **q-PCR, Cloning of the 84aas CRNDEP ORF into 3 expression vectors, Western blot, Immunoprecipitation of the CRNDEP peptide, Cellular localization studies using fluorescence microscopy, IHC staining of the CRNDEP peptide in human tissues, Detection of endogenous CRNDEP in HeLa cells by**  **Immunofluorescence, shRNA-mediated knockdown of CRNDE** | **peptide produced by the CRNDE gene called CRNDEP can be present in the structure of SGs and is overexpressed in highly proliferative tissues.** |
| **Grabocka et al. 2016** | **USA** | **Cell culture**  **Animal study**  **Human Pancreasample** | **Six pancreatic adenocarcinoma and three normal**  **tissues adjacent to PDACs** | **NCr nude mice** | **DLD1**  **HT-29**  **NCI-H747**  **NCI-H508**  **SNUC-1**  **Mia-PaCa-2**  **Panc-1**  **AsPC1**  **Capan2**  **Hs700T**  **HEK293T**  **HeLa cells** | **G3BP1**  **eIF4G** | **Immunofluorescence and IHC, Quantification of stress granules, q-PCR, FACS analysis, ELISA, Proliferation, Analysis of human patient data** | **In K-RAS mutant tumor cells, the presence of SGs is increased by increasing the production of 15-d-PGj2 (a lipid compound).**  **Up-regulating SGs causes resistance to stress.** |
| **Valentin-Vega et al. 2016** | **USA** | **Cell culture** | **-** | **-** | **HEK293T** | **DDX3X**  **G3BP1**  **G3BP2**  **eIF4G** | **Cys metabolic labeling, Puromycin incorporation assay, RNA-seq, CLIP-seq, Ribosome profiling** | **Cancer-related DDX3 mutations can change the condition by stopping the translation process globally to cause cancer cell survival.** |
| **Gupta et al. 2016** | **USA** | **Cell culture**  **Animal study** | **-** | **NOD-SCID mice** | **BT-474**  **MDA-MB-231**  **MDA-MB-453** | **G3BP2**  **PABP1** | **Flow Cytometry, Mammosphere Assay, Xenograft Tumor Transplantation and ELDA, Survival Analysis** | **the mRNA molecule of The squamous cell carcinoma antigene by T cell-3 (SART3) is stabilized by G3BP2 and can increase the expression of pluripotency transcription factors OCT4 and NANOG and regulates breast tumor initiation.** |
| **Weeks et al. 2016** | **Canada** | **Cell culture** | **-** | **-** | **U251**  **U343**  **U118**  **SNB19**  **A341**  **U87**  **T98**  **293T** | **TIAR G3BP1** | **IHC, Coimmunoprecipitation,** **siRNA Transfection, RNA Immunoprecipitation,**  **Cellomics Assay,**  **Microarrays** | **AurkB and ECT-2 are located in the structure of SGs in astrocytoma cells.**  **Inhibition of AurkB reduces the number of SGs. SGs in astrocytoma cells contain important mRNAs related to metabolism, translation, and cell migration.** |
| **Wall et al. 2016** | **Canada** | **Cell culture** | **-** | **-** | **HeLa cells**  **U2OS** | **hnRNPA1**  **PRMT1** | **Western blot, Cellular translation assay, Immunoprecipitation, q-PCR, Microscopy** | **hnRNPA1 is a component of SGs in which methylarginines within the RGG-motif region are essential for the function of the IRES trans-acting factor.**  **Hypomethylation and mutation of the RGG motif can involve it in cancer.** |
| **Chiou et al. 2017** | **China** | **Cell culture**  **Human CRC sample** | **one stage IIA and two stage IIB**  **samples and three normal samples** | **-** | **HT-29**  **HCT-116** | **MSI1**  **PABP1**  **eIF4E** | **Plasmid constructions and small interfering RNA knockdown, Spheroid Formation and Transwell Assay,** **Immunoblotting and Immunofluorescence Staining,**  **FITC-gelatin Degradation Assay,** **Clonogenic Assay,** **Flow Cytometry Assay,** **TUNEL Staining** | **MSI1 promotes the development of CD44 cancer stem cells in colorectal cancer and promotes resistance to chemotherapy by forming MSI1 associated SGs.** |
| **Coppin et al. 2017** | **France** | **Cell culture**  **Human**  **pancreatic sample**  **Animal study** | **Ten pancreatic tumour samples** | **Gal-3 null mutant mice** | **CAPAN-1**  **pancreatic cancer cells** | **hnRNP-L**  **Gal-3** | **Reversion experiment and Gal-3 Inhibitor treatment, q-PCR, Western Blot, RNA-IP and RNA-Seq, FISH and imaging studies, Proximity ligation assay, RNA Pull Down, IHC, Coimmunoprecipitation** | **Gal-3 acts as a non-classic RBP by interaction with hnRNP-L in the structure of SGs.**  **Gal-3 in the structure of hnRNP-L-containing RNA granules stabilizes MUC4 mRNA in the cytoplasm of cancer cells.** |
| **Morettin, A.**  **et al. 2017** | **Canada** | **Cell culture**  **Animal study** | **-** | **SHC mice**  **(n = 3)**  **SHO mice (n = 3)** | **293T**  **MDA MB 231**  **Hs578T**  **MCF-7** | **TDRD3** | **MTT Assay, Cell Motility and Invasion Assays, In Vivo Mouse Experiments, Xenograft Mice Experiments, Tail Vein Injections, Immunoblotting, q-PCR, Sucrose Gradient Fractionation, Chromatin Immunoprecipitation, RNA Immunoprecipitation** | **TDRD3, as a vital member of the protein components of cytoplasmic SGs, plays an essential role in tumorigenesis and invasion in breast cancer cells.**  **Cell depletion of TDRD3 prevents the formation and progression of cancer cells.** |
| **Narayanan et al. 2017** | **USA** | **Cell culture** | **-** | **-** | **VMRC-LCD cells**  **MDA-MB-231**  **HeLa cells**  **HEK293** | **TDRD3**  **USP9X**  **PRMT1**  **TIAR**  **G3BP1** | **GST pull-down, Immunoprecipitation of arginine-methylated proteins, In vivo ubiquitination, In vitro methylation, Immunofluorescence, q-PCR, Apoptosis assay** | **TDRD3 and USP9X are colocalized together in the structure of cytoplasmic SGs.**  **TDRD3 is required to protect USP9X from de-ubiquitination.**  **TDRD3 Knocking down sensitizes breast cancer cells to chemotherapy and increases apoptosis during the inhibition of USP9X localization in the SGs structure.** |
| **Chen et al. 2018** | **China** | **Cell culture**  **Animal study** | **-** | **Nude mice** | **U87**  **U251** | **MSI1**  **eIF2α** | **IHC,**  **Western blot, cell viability assay** | **MSI1 equips cancer stem cells and increases chemoresistance in glioblastoma cells By affecting the PKR / eIF2α pathway and forming SGs.** |
| **Takayama et al. 2018** | **Japan** | **Cell culture** | **-** | **-** | **VCaP**  **293T** | **G3BP2**  **USP10** | **Immunoblot analysis and immunoprecipitation,Cell proliferation assay, Immunofluorescence,**  **IHC,**  **q-PCR** | **USP10 expression is elevated in prostate cancer cells.**  **Interaction with G3BP2 inhibits the P53 signaling pathway and induces a specific oncogenic effect in prostate cancer cells along the USP10 / G3BP2 / P53 pathway.** |
| **Wang et al. 2018** | **China** | **Cell culture**  **Human RCC sample**  **Animal study** | **43 pairs of primary RCC tissues**  **43 para-carcinoma**  **normal tissues** | **Nude mice** | **A498**  **ACHN**  **293T** | **G3BP1** | **Western blot,** **IHC, Cell proliferation assay,** **Cell migration assay,** **Tumor cell invasion assay,** **Chemotaxis assay,** **Pathway dual-luciferase reporter assay,** **Xenograft tumor growth and metastasis** | **G3BP1 expression is increased in tumor cells.**  **G3BP1 promotes tumor progression and metastasis in renal cell carcinoma cells by acting in the IL6 / G3BP1 / STAT3 pathway.** |
| **Choi et al. 2019** | **South Korea** | **Cell culture**  **Human colon cancer sample**  **Animal study** | **colon cancer and normal tissue** | **C57BL/6 J mice** | **HeLa cells** | **Rbfox2** | **Flow cytometry, Immunoprecipitation and immunoblot analysis,** **Immunofluorescence microscopy,** **RBP immunoprecipitation (RIP) polymerase chain reaction**  **(PCR),** **Reverse phase protein microarray (RPMA)** | **Rbfox2 in the structure of SGs increases the proliferation process in cells by affecting the expression of RB1 and reducing it.**  **By isolating Rbfox2 from the structure of SGs, resveratrol inhibits the action of Rbfox2 on RB1 and reduces cancer overgrowth.** |
| **Haghandish et al**. 2019 | **Canada** | **Cell culture** | **-** | **-** | **MCF-7**  **MDA-MB-231**  **HEK293T** | **PRMT7**  **eIF2α**  **FMRP**  **TIAR**  **eIF2β**  **eIF4G**  **eIF2γ** | **Production of lentivirus and cell transduction/Fluorescence microscopy/SILAC-based affinity purification and mass spectrometry/Coimmunoprecipitation/Polyribosome profiling/Protein purification/Methylation assays/Immunofluorescence - Stress Granules** | **At different cellular stresses, PRMT7 can methylate eIF2α in breast cancer cells.**  **The presence of PRMT7 is required for the phosphorylation of eIF2α in residue serin51 in the process of stress-dependent granule formation.** |
| **Heberle et al. 2019** | **Netherlands** | **Cell culture** | **-** | **-** | **MCF-7**  **HEK293T** | **S6K1**  **S6K2**  **eIF2α**  **G3BP1** | **Cell lysis and immunoblotting,**  **Immunofluorescence,**  **RAS assay** | **Translation pathway enhancers, MAPK / P38, were identified as pro-stress granule kinases.**  **PI3K and P38 drive MTORC1 activity to lead to SG assembly and accelerate SG formation.** |
| **Kashiwagi et al. 2019** | **Japan** | **Cell culture** | **-** | **-** | **Hela cells**  **Cos-1**  **K562**  **Ku812**  **TOM-1**  **ALL/MIK**  **MY**  **WEHI-3**  **Ba-F3/CL1** | **HSP90α**  **DCP1a** | **Live-cell imaging, Immunofluorescence, Proliferation assay, Cell viability assay** | **Bcr-Abl is colocalized in the structure of SGs. This colocalization is involved in granule structure in Bcr-Abl dependent leukemogenesis.** |
| **Lin et al. 2019** | **China** | **Cell culture**  **Human gasteric cancer sample** | **167**  **Human gasteric cancer sample** | **-** | **SGC7901**  **BGC823**  **MGC803**  **MGC803**  **MKN45** | **ATXN2L**  **G3BP1** | **Western blot, Immunofluorescence, q-PCR, ROS detection and cell apoptosis assays, Cell viability, proliferation, and colony formation assays, Migration and invasion** | **EGF can up-regulate Ataxin-2-like (ATXN2L), a regulator of stress granules, in the PI3 / AKT signaling pathway.**  **This up-regulation results in oxaliplatin resistance and ultimately increase cell invasion in gastric cancer.** |
| **Mazloomian et al. 2019** | **Canada** | **Cell culture** | **-** | **-** | **Hela cells**  **HCT1-116** | **G3BP1**  **GRB2**  **TIA1**  **YB-1** | **Transfection of siRNAs, Apoptosis assay, Immunofluorescence/Cell cycle analysis by flow cytometry, Alignment and calculating gene and isoform expression, Alternative splicing analysis, Clustering of expression responses, Stress granule induction and detection** | **eIF4A3 is one of the pillars in the biology of SGs, which can increase the survival of cancer cells and tumor progression by sequestration and silencing the translation process with a significant impact on TIA1 and G3BP1.** |
| **Zheng et al**. 2019 | **China** | **Human tissue specimens** | **247 cases of NSCLC**  **48 noncancerous lung tissues** | **-** | **-** | **G3BP1**  **YB-1** | **Patient cohorts and tissue microarrays**  **(TMAs), IHC** | **G3BP1 is a prognostic biomarker in NSCLC patients due to its essential role in activating the PI3K / AKT / mTOR pathway.** |
| **Cui et al. 2020** | **USA** | **Cell culture** | **-** | **-** | **U2OS** | **DDX3**  **G3BP1** | **image acquisition, processing, and analysis, Immunofluorescence staining, siRNA transfection, Western blot** | **DDX3 is an essential target for the development of cancer treatment.**  **The effect of DDX3 was inhibited by two molecular inhibitors, RK33 and 16D. DDX3 was also knocked down as RNA-mediated, resulting in a significant reduction in SG assembly.** |
| **Do et al. 2020** | **Canada** | **Cell culture** | **-** | **-** | **U2OS**  **MCF-7**  **HEK293** | **TIA1**  **G3BP2** | **CRISPR, Cas 9 gene editing, RT-qPCR,**  **Metabolic labeling, Preparation of cell extracts and affinity purification, Mass spectrometry and data analysis, Fluorescence microscopy, Expansion microscopy** | **The formation of SGs in response to stress in cancer is one of the main pro-survival pathways for therapeutic targeting.**  **Intranuclear parallel pathways can also be significant pathways against stress in cancer, including the accumulation of RepoMan in nucSF in the nucleus.** |
| **Vellky et al. 2020** | **USA** | **Cell culture**  **Human prostate cancer sample**  **Animal study** | **14 Human prostate cancer sample** | **athymic Nu/Nu mice** | **BCaP cell**  **LAPC4 cell** | **DDX3**  **PABP1** | **RNA Immunoprecipitation, In Situ Hybridization, and qPCR, DDX3 Overexpression and Inhibition,** **Renal Capsule Xenografts** | **In castration-resistant prostate cancer (CRPC) cells, DDX3 acts as a post-transcriptional regulation mechanism for the androgen receptor (AR) and can be a target for AR-low / CRPC sensitization to AR-directed therapies.** |
| **Zhao**  **et al. 2020** | **China** | **Cell culture**  **Human gastric cancer sample**  **Animal study** | **fifty-five gastric cancer patient samples** | **Balb/c nude**  **mice** | **MGC80-3**  **HGC-27**  **SGC-7901**  **BGC-823** | **PARP1**  **G3BP1**  **YWHAZ**  **eIF4D** | **IHC,**  **Western blotting,** **Mitochondria isolation,** **Cytotoxicity assay,** **Cell apoptosis and cell cycle assay,** **Colony formation assay,** **Confocal microscopy,** **Co-immunoprecipitation,** **Tumorigenesis in xenograft mouse model** | **G3BP1, as a significant component of SGs in gastric cancer, is overexpressed. Silencing and emptying the cell of G3BP1 leads the cell to apoptosis and increases sensitivity to chemotherapy.**  **G3BP1 has high interaction with YWAHZ, and G3BP1highYWHAZhigh patients have the worst outcome compared to other patients.** |
| **Brown et al. 2021** | **USA** | **Cell culture** | **-** | **-** | **yeast** | **DDX3X** | **Western blotting,**  **Polyribosome preparation,**  **Scanning assays,**  **Fluorescence microscopy,**  **ATPase activity assays,**  **RNA binding assays** | **Induction of SG formation occurs by**  **DDX3X / DED1 mutations associated with medulloblastoma and is associated with defects in the translation process.** |
| **Wang et al. 2021** | **China** | **Cell culture**  **Animal study** | **-** | **Balb/c nude mice** | **HCT116**  **HT29**  **HEK293T**  **NCM460** | **G3BP1**  **CAPRIN1** | **Cell culture and lentiviral transduction, q-PCR,**  **Absolute RNA quantitation,**  **Metabolic measurements,**  **GFP reconstitution assay,**  **RNA in situ hybridization and immunofluorescence,**  **Western blotting and IPs,**  **RNA immunoprecipitation,**  **Luciferase reporter assay,**  **Xenograft model** | **In the absence of glutamine, an increase in GIRGL LncRNA levels in the cell causes a complex between GLS1 mRNA and CAPRIN1.**  **On the other hand, increasing the LLPS process in CAPRIN1 induces SGs, and inhibition of GLS1 mRNA translation increases the survival of cancer cells.** |
| **Zhang et al. 2021** | **USA** | **Cell culture**  **Human breast cancer sample**  **Animal study** | **47**  **Human breast cancer sample** | **FVB/N mice** | **MCa-PSTC**  **CT2A** | **G3BP2** | **Western blotting,**  **qRT-PCR,**  **Immunofluorescence assay,**  **RNA immunoprecipitation assay, IHC,**  **Flow cytometry** | **Expression of G3BP1 and PD-L1 in cancer tissues also showed high co-expression.**  **Knockdown or silencing of G3BP2 by c108 also decreased PD-L1 expression due to increased mRNA degradation.** |
